# Computational Insights and In Silico Characterization of a Novel Mini-Lipoxygenase from Nostoc Sphaeroides and Its Application in the Quality Improvement of Steamed Bread

**DOI:** 10.3390/ijms24097941

**Published:** 2023-04-27

**Authors:** Bingjie Xia, Huibing Chi, Bingjie Zhang, Zhaoxin Lu, Huawei Liu, Fengxia Lu, Ping Zhu

**Affiliations:** College of Food Science and Technology, Nanjing Agricultural University, Nanjing 210095, China

**Keywords:** *Nostoc sphaeroides*, lipoxygenase, catalytic activity, superior affinity, stability

## Abstract

Lipoxygenase (EC1.13.11.12, LOX) has been potentially used in the food industry for food quality improvement. However, the low activity, poor thermal stability, narrow range of pH stability, as well as undesirable isoenzymes and off-flavors, have hampered the application of current commercial LOX. In this study, a putative mini-lipoxygenase gene from cyanobacteria, *Nostoc sphaeroides* (NsLOX), was cloned and expressed in *E. coli* BL21. NsLOX displayed only 26.62% structural identity with the reported LOX from *Cyanothece* sp., indicating it as a novel LOX. The purified NsLOX showed the maximum activity at pH 8.0 and 15 °C, with superior stability at a pH range from 6.0 to 13.0, retaining about 40% activity at 40 °C for 90 min. Notably, NsLOX exhibited the highest specific activity of 78,080 U/mg towards linoleic acid (LA), and the kinetic parameters—*K*_m_, *k*_cat_, and *k*_cat_/*K*_m_—attain values of 19.46 μM, 9199.75 s^−1^, and 473.85 μM^−1^ s^−1^, respectively. Moreover, the activity of NsLOX was obviously activated by Ca^2+^, but it was completely inhibited by Zn^2+^ and Cu^2+^. Finally, NsLOX was supplied in steamed bread and contributed even better improved bread quality than the commercial LOX. These results suggest NsLOX as a promising substitute of current commercial LOX for application in the food industry.

## 1. Introduction

Lipoxygenases (EC1.13.11.12, LOXs), as an assemblage of non-heme iron or manganese-containing dioxygenases, can catalyze the stereospecific dioxygenation of polyunsaturated fatty acids (PUFAs) containing one or more Z, Z-1, or 4-pentadiene structures into hydroperoxy fatty acids (HpFAs) [1]. LOXs have become increasingly appealing in pharmaceutical and food industries. Particularly, they have been confirmed to be a safe and friendly natural additive in food processing to modify the strength of dough by altering the cross-linking of the gluten network, unifying the color value of wheat flour by providing a bleaching effect through carotene oxidation, and promoting volatile flavor formation of food by catalyzing the oxidation of linoleic and linolenic acids [2]. Considering their crucial roles in the food industry, LOXs with desirable properties have drawn great attention in facilitating their application.

Currently, LOXs from animals and plants have been extensively investigated. Based on the hydroperoxide-forming activity at specific carbon positions toward fatty acids or triglyceride methyl esters, mammalian LOXs with molecular masses of 75–80 kDa are mainly classified as 5-, 8-, 12-, and 15-LOX by catalyzing long chain PUFAs (such as arachidonic acid (AA) and docosahexaenoic acid (DHA)) [3]. Differently, plant LOXs with larger molecular masses of 94–104 kDa are classified as 9-LOX and 13-LOX using C18 PUFAs (such as linoleic acid (LA), α-linolenic acid (ALA), and γ-linolenic acid (GLA)) as substrate [4]. Amongst these two sources, LOXs from a soy source are mainly served for commercial applications [5]. However, the isolation of LOXs from soy has the disadvantage of season-dependent growth [2]. In addition, the by-components from soy extraction cause undesirable effects, including the reduced availability of PUFA substrates and turnover of LOXs due to the presence of soy isoenzymes [6], and the formation of off-flavors is associated with other components from soy [7]. Thus, it is desirable to explore other alternative sources of LOX to meet the great demands for its application in the food industry.

Microbial LOXs have been recognized for their commercial potential due to the high yield and low cost of industrial production [8]. Among them, LOXs from *Pseudomonas aeruginosa* [9], *Anabaena* sp. [10], *Myxococcus xanthus* [1], *Enterovibrio norvegicus* [11], and *Nostoc* sp. [12] have been currently characterized. Nevertheless, the physiological roles and metabolites of microbial LOXs, as well as those of plant and mammal LOXs, are limitedly investigated. More importantly, the disadvantages of low affinity to substrates, poor thermal stability, low catalytic efficiency, and potential risk of disease limit the industrial application of microbial LOXs.

Therefore, it is necessary to hunt for new microbial LOXs to alleviate these issues. In this present study, a novel LOX gene from *N. sphaeroides* (NsLOX) was successfully cloned and recombinantly expressed in *Escherichia coli* BL21. Besides the purification and characterization of NsLOX, its catalytic activity on the quality of steamed bread was compared with commercial soybean LOX. To the best of our knowledge, this is the first study to explore the characteristics of LOX from a *N. sphaeroides* source.

## 2. Results and Discussion

### 2.1. Excavation of a Hypothetical LOX from N. sphaeroides

**Figure 1:**
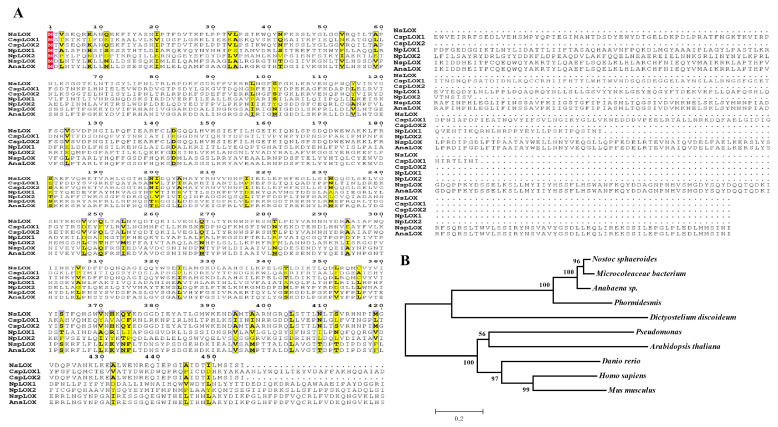
(**A**) Sequence alignment of NsLOX with other cyanobacterial LOXs. Highly conserved residues are shown with a yellow background, while strictly conserved residues are shown with a red background. (**B**) Phylogenetic tree analysis of amino acid sequences of LOXs using the N-J method by MEGA 5.0 software.

Firstly, the hypothetical LOX gene from the *N. sphaeroides* strain was obtained from the NCBI website. The open reading frame (ORF) of the NsLOX gene was 1368 bp in length, encoding for 455 amino acid residues, with the theoretical molecular weight and isoelectric point of 52 kDa and 5.84 found by ProtParam tool (https://web.expasy.org/protparam/, accessed on 7 October 2022). Then, the primary structure identity of NsLOX (WP_118168638.1) was compared with 6 characterized cyanobacterium LOXs, including CspLOX1 (WP_012595715.1) of 668 amino acid residues and CspLOX2 (WP_118168638.1) of 569 amino acid residues from *R. orientalis* PCC 8801 [13], NpLOX1 (ZP_00106490.1) of 630 amino acid residues and NpLOX2 (ZP_00107030.1) of 547 amino acid residues from *Nostoc punctiforme* PCC 73102 [14,15], NspLOX (WP_010994078.1) of 773 amino acid residues from *Nostoc* sp. SAG 25.82 [12], and AnaLOX (WP_010994078.1) of 773 amino acid residues from *Anabaena* sp. PCC 7120 [10] (Figure 1A). To be noted, the amino acids of NsLOX were less and shared lower identities with other LOXs, indicating that they belonged to the group of prokaryotic mini-LOXs (a rare group of LOX enzymes found in a few bacteria with low sequence homology to all characterized LOXs). Further, the phylogenetic analysis of LOXs from plant (*Arabidopsis thaliana*), animal (*Homo sapiens*, *Mus musculus*, *Dictyostelium discoideum*, and *Danio rerio*), bacteria (*Pseudomonas*), and cyanobacteria (*Microcoleaceae bacterium*, *Phormidesmis*, and *Anabaena* sp.) are illustrated in Figure 1B. Surprisingly, NsLOX was closely clustered with LOXs of cyanobacteria, but it was greatly separated from LOXs of animals, plants, and bacteria, possessing NsLOX of diffident functions and substrate specificity in the biotransformation of PUFAs compared with those of animals, plants, and bacteria (Figure 1B).

### 2.2. Structural Modeling of NsLOX and Structure Analysis

**Figure 2:**
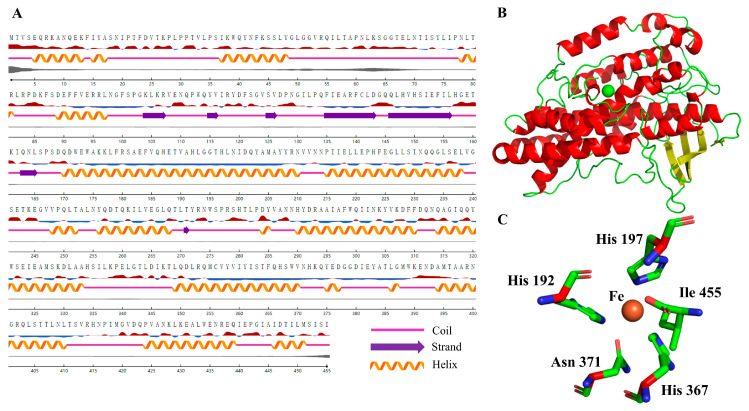
(**A**) Secondary structure prediction for the deduced amino acid sequence of a NsLOX gene from *N. sphaeroides* by NetSurfP-2.0. (**B**) The three-dimensional model prediction of NsLOX from *N. sphaeroides*. (**C**) The catalytically active center of NsLOX.

Generally, the 3D structure is crucial for enzyme activity, stability, catalytic efficiency, and substrate specificity. Firstly, the secondary structure of NsLOX was predicted using NetSurfP-2.0 (Figure 2A). The result showed that it contained 18 α-helices, 7 β-strands, and a large number of coil structures, while lacking N-terminal structure, which was presented in other LOXs [16]. To further analyze the overall structural differences between NsLOX and other LOXs, the 3D structure of NsLOX was predicted using the LOX of *Cyanothece* sp. (PDB ID: 5EK8, structural identify: 26.62%) as a template by the Swiss-Model server (Figure 2B). Notably, NsLOX exhibited a quite low structural identity with other LOXs. Moreover, NsLOX was considered as a monomer, which was different from dimer structures of the eukaryotic LOXs. Interestingly, NsLOX mainly consisted of 50% α-helix, 11% β-strand, and 38% coil, of which the α-helix content in NsLOX was significantly higher than that in *Cyanothece* sp. LOX (PDB ID: 5EK8, 40% α-helix) [17], *Glycine max* LOX (PDB ID: 1YGE, 36% α-helix) [18], and *Plexaura homomalla* LOX (PDB ID: 2FNQ, 32% α-helix) [19]. Studies suggest that the amount of α-helix has a positive correlation with protein stability [20,21], this high percentage of α-helix suggests that NsLOX might have better thermal stability. The catalytically active center of NsLOX involved non-heme iron three conserved histidine residues (His192, His197, His367), Asn371, and C-terminal Ile455 (Figure 2C), which were conserved and consistent in most LOXs [16,22]. These overall structure comparisons between NsLOX and other LOXs preliminarily indicated NsLOX as a novel LOX.

**Figure 3:**
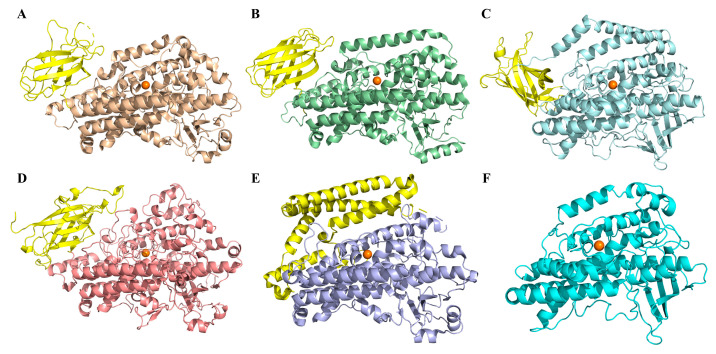
Comparison of the N-terminal domain of NsLOX with those of other LOXs. (**A**) *P. homomalla* LOX, PDB ID: 2FNQ (**B**) *Oryctolagus cuniculus rabbit* LOX, PDB ID: 2P0M. (**C**) *Cyanothece* sp. LOX, PDB ID: 5EK8. (**D**) *G. max* LOX, PDB ID:1YGE. (**E**) *P. aeruginosa* LOX, PDB ID: 5LC8. (**F**) NsLOX in this study.

Beyond clearly different overall structure, the specific local structure of NsLOX was also significantly different from other LOXs. So far, LOX could be classified into three groups based on the structure of the N-terminal domain. The first group was regarded as the N-terminal of a LOX fused with other enzymes, such as *P. homomalla*, and *Anabaena* sp. LOXs having a fused allene oxide synthase in their N-terminals (Figure 3A) [10,19]. The second group was the typical LOX with a β-barrel N-terminal domain (PLAT domain) consisting of multiple, and inversely parallel, β-sheets of a molecular weight of about 25–30 kDa. The majority of LOXs from mammals (Figure 3B), bacteria (Figure 3C), and plants (Figure 3D) belong to this group. The third group was the LOX with the α-helix N-terminal domain, forming a cap over the substrate binding region. *P. aeruginosa* LOX was the only one that belonged to this group (Figure 3E) [9,23]. Related studies have revealed that lack of PLAT domain of LOX does not affect its catalytic activity and other enzymatic properties, including the Michaelis constant (*K*_m_), maximum rate of reaction (*V*_max_), optimum pH, and substrate specificity [16,24,25,26,27]. Moreover, the removal of the N-terminal domain of LOX was presumed to facilitate the penetration of the substrate into the catalytic pocket and the release of the product from the active site of the enzyme [28,29]. Interestingly, NsLOX had lacked the N-terminal domain, which was quite different from the above three groups (Figure 3F). Therefore, NsLOX was confirmed as a novel and unique LOX with catalytic activity for food industry application.

### 2.3. Expression and Purification of NsLOX in E. coli

**Figure 4:**
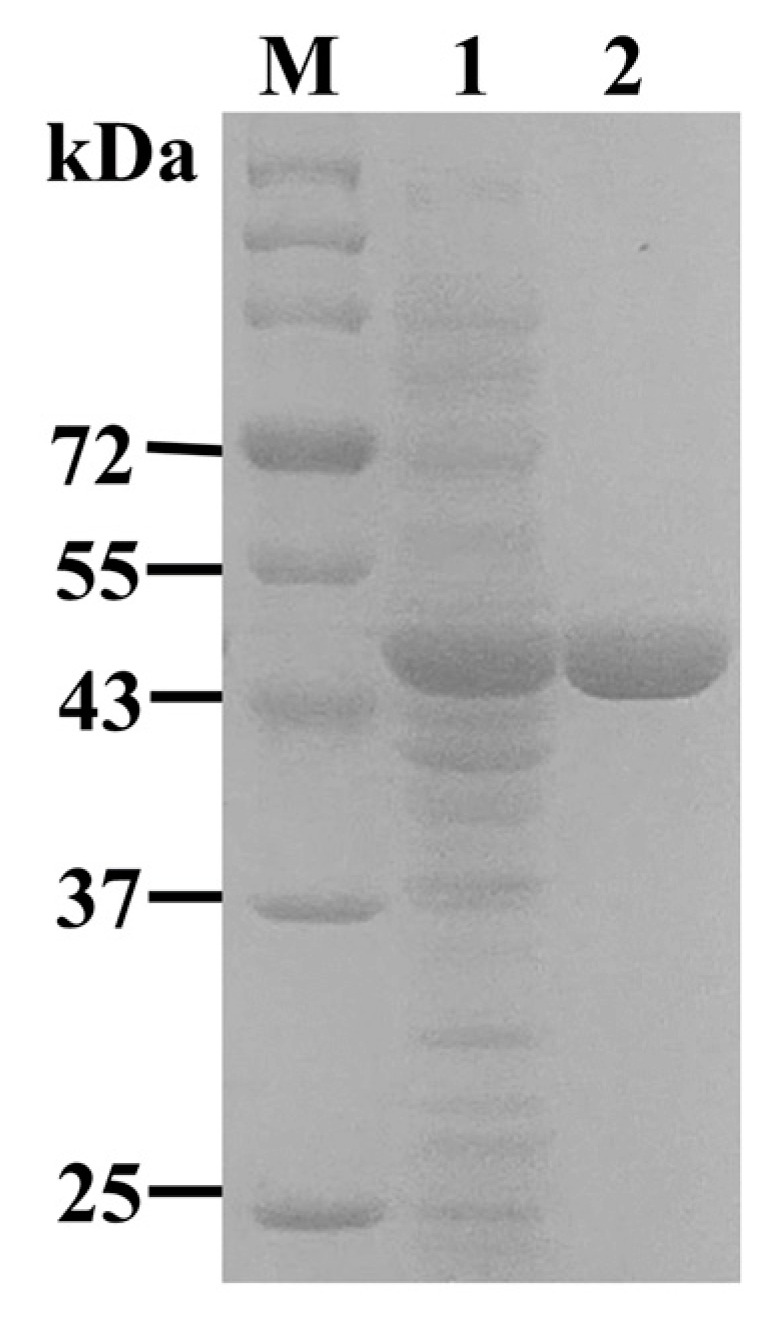
The SDS-PAGE analysis of NsLOX expression and purification. M: Marker 26616. Lane 1: cell-free enzyme of NsLOX. Lane 2: purified NsLOX.

In order to determine the properties, NsLOX was over-expressed in the *E. coli* BL21 (DE3) host. After cell lysis, the activity towards LA of NsLOX reached 6728 ± 180.13 U/mL, with an apparent band of about 54 kDa containing NsLOX (52 kDa) and tag (2 kDa) in SDS-PAGE (Figure 4, Table 1), which displayed similar molecular mass to that of *Nostoc* sp. LOX of 52 kDa [12]. However, it was much lower than LOXs from plants and animals (75–104 kDa) and slightly higher than LOX from *N. punctiforme* (45 kDa) [15]. The specific activity toward the LA of NsLOX (78,080 U/mg) was 47.38 times higher than that of the crude enzyme (Table 1), which was particularly much higher than that of LOXs from *Anabaena* sp. PCC 7120 (30,000 U/mg) [30] and *M. xanthus* (56,000 U/mg) [1]. Since the lower specific activity need costs more for industrial applications, NsLOX with high specific activity, in our study, could overcome this shortcoming.

### 2.4. Enzymatic Properties of the Purified NsLOX

**Figure 5:**
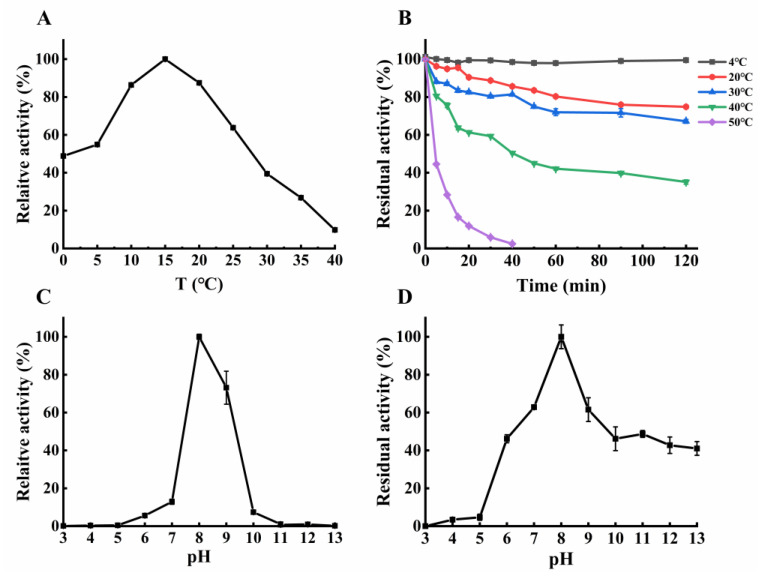
The enzymatic properties of purified NsLOX. (**A**) Effects of temperatures on the catalytic activity of NsLOX. (**B**) Effects of temperatures on the pH stability of NsLOX. (**C**) Effects of pH values on the catalytic activity and stability of NsLOX. (**D**) Effects of pH on the thermostability of NsLOX. Means ± SD were obtained from three independent measurements.

As temperature and pH are the most key parameters in food processing, the optimum reaction temperature and thermostability of NsLOX were firstly assessed by measuring the relative and residual activities of LOX under different temperatures. NsLOX displayed 80% relative activity from 10 to 20 °C, with the optimal temperature of 15 °C (Figure 5A), which was similar to most LOXs (15–25 °C) [31] but significantly lower than that of *G. avenae* LOX (60 °C) [32]. To look into the thermal stability, the residual activity of NsLOX was evaluated after keeping it at different temperatures (Figure 5B). Its residual activity remained unchanged after 120 min storage at 4 °C. As the temperature increased to 20 and 30 °C, NsLOX retained more than 65% residual activity after 120 min treatment. More than 40% of residual activity remained after 90 min treatment at 40 °C. However, when the temperature increased to 50 °C, the residual activity decreased rapidly and only remained over 40% activity after 5 min treatment. Compared to *Anabaena* sp. LOX (t_1/2_ = 2.3 min at 50 °C) [10], NsLOX exhibited high thermal stability, which was also consistent with our structural analysis.

To explore the optimum pH and pH stability of NsLOX of NsLOX, the relative and residual activities of LOXs, under different pHs, were investigated in Figure 5C,D. Generally, the optimal pH for most LOXs was 7.5–9.0 [31,33], NsLOX showed maximum activity at pH 8.0, and it even still had 60% activity at pH 9.0, which was in agreement with most LOXs (Figure 5C), but it was different from the *Camellia sinensis* LOX of the highest activity at pH 3.6 [34] and the *M. xanthus* LOX of he highest activity at pH 3.0 [1]. Surprisingly, NsLOX exhibited excellent stability over a wide pH, ranging from 6.0 to 13.0, with over 40% residual activity at pH 6.0–13.0 after 24 h incubation (Figure 5D). Compared with considerable LOXs of better stability at pH 4.5–8.0 [6], NsLOX was super stable, under both acidic and alkaline conditions, for its industrial application.

Since improving catalytic efficiency is beneficial for the economic production of enzymes on an industrial scale, adding metal ions and compounds is regarded as one key approach. Thus, the effects of various compounds and metal ions on NsLOX activity were investigated in Table 2. Among these, Ca^2+^ and Mg^2+^ could activate the catalytic activity of NsLOX with increased activity of 167.91% and 39.81%, respectively, which had concentration-dependent properties and acted as co-factors by firstly activating the substrate and, then, completing the catalytic reaction. This is similar to the nature of other LOXs such as EnLOX (136.8%) [11] and MxLOX (59%) [1]. The presence of Ca^2+^ binding sites at the N-terminal end of some LOXs has been reported in the literature, which correlates with the intracellular regulation of LOX activity molecular levels [35,36]. However, Fe^2+^, Fe^3+^, and Mn^2+^ inhibited the activity of NsLOX with reduced activity of 76.01%, 16.20%, and 48.40%, respectively, which was in agreement with the findings of *P. aeruginosa* LOX [9] and *E. norvegicus* LOX [11] displaying completely inhibited activity with increased ion concentrations. Zn^2+^, Cu^2+^, EDTA, and SDS could completely inhibit the activity of NsLOX, even at low concentrations. Cu^2+^ affects the oxylipin blend and, thus, inhibits enzyme activity [37]. These results suggested that Mg^2+^ and Ca^2+^ could be introduced into the late fermentation process to optimize the fermentation conditions of NsLOX.

### 2.5. Substrate Spectrum, Kinetic Parameter, and Product Analysis of Purified NsLOX

The substrate specificity of NsLOX was evaluated by comparing the relative activities against different PUFAs (Table 3). NsLOX showed the highest activity towards LA, a higher relative activity towards ALA (83.12%), and high relative activity towards AA (65.57%). LA was the optimal substrate for NsLOX, which was consistent with that of *C. sinensis* LOX [34], *N. punctiforme* LOX [38], and *E. norvegicus* LOX [11].

The kinetic parameters, including *K*_m_, *V*_max_, and *k*_cat_ for NsLOX on LA, ALA, and AA, were calculated from Lineweaver–Burk double inverse plots (Table 4). The *K*_m_ and *k*_cat_/*K*_m_ values of NsLOX for LA were 19.46 μM and 473.85 μM^−1^ s^−1^, whereas the *K*_m_ values for ALA and AA were 150.94 and 571.22 μM, and *k*_cat_/*K*_m_ values were 62.13 and 12.79 μM^−1^ s^−1^, respectively, further confirming that LA was the optimal substrate of NsLOX (Table 4). NsLOX had higher LA affinity than those of *C. sinensis* LOX (141 μM) [34], *Cucumis melo var. akuwa* LOX (126 μM) [39], *A. aegerita* LOX (296 μM) [33], and *P. aeruginosa* LOX (660 μM) [9]. Moreover, the catalytic efficiency of NsLOX towards LA was also higher than those of *P. aeruginosa* LOX (38.55 μM^−1^ s^−1^) [8], *E. norvegicus* LOX (4.83 μM^−1^ s^−1^) [11], and *C. sinensis* LOX (38.55 μM^−1^ s^−1^) [34]. LOX catalyzes the reaction of linoleic acid by binding an oxygen molecule to the 9- or 13-position of the carbon chain of linoleic acid. The C atom attached to the conjugated double bond on the carbon chain of linoleic acid undergoes dehydrogenation, the hydrogen atom reduces the trivalent iron in the active center to divalent iron, the dioxygen atom mediates the insertion from the side of the hydrogen extraction site, and the C atom with free electrons undergoes a double addition of oxygen to form a peroxy radical, which is then reduced, by electrons, from divalent iron to the corresponding anion, and the divalent iron is re-oxidized to form trivalent iron [40]. The product of 9/13-LOX is reduced to produce 9-HODE and 13-HODE [4]. To determine the classification, the products of LA by NsLOX catalysis were identified by LC-MS (Figure S1). The two main peaks at 9.09 and 9.17 min corresponded to the retention times of the standards of 9-HODE and 13-HODE, respectively, suggesting that NsLOX could be defined as 9/13-LOX. Taken together, the good affinity for LA, the extremely high catalytic efficiency, and the suitable classification of NsLOX revealed NsLOXs of great potential application values in the food industry.

### 2.6. Application of Purified NsLOX in the Steamed Bread

In order to verify the activities of NsLOX and the current commercial LOX on the steamed bread, the quality structures of steamed bread supplied with LOXs were examined separately by the texture profile analysis (TPA) test. The TPA test is used to quantify the hardness, springiness, gumminess, and chewiness of the sample by simulating the chewing behavior of the teeth, as well as the changes in time and force during the secondary chewing process. Thus, all these parameters were verified for both NsLOX and commercial LOX treatments (Table 5). The hardness, gumminess, and chewiness of the groups containing NsLOX were significantly different from those of blank and commercial LOX groups (*p* < 0.05). It has been well documented that LOX confers to improved flour strength by oxidizing sulfur groups in gluten proteins [41], and the decreasing of hardness, stickiness and chewiness gave bread a better quality as the gluten strength increased [30,42,43]. NsLOX led to the lowest hardness, gumminess, and chewiness, indicating that it contributed to the super improvement of the steamed bread. The springiness of the steamed bread by NsLOX was not significantly changed from the blank group, which is similar to the study of *G. max* LOX and superior to Analox [42,44]. Moreover, there were no significant differences of springiness between NsLOX and commercial LOX treatments, implying that NsLOX displayed favorable features compared with commercial LOX as a flour improver.

## 3. Materials and Methods

### 3.1. Strains and Chemicals

The cDNA sequence of NsLOX gene was downloaded from NCBI database (Genbank no. NZ_CP031941.1: D1367_RS23555). The codon of NsLOX was optimized according to the corresponding codon of *E. coli* BL21 by JCAT website (http://www.jcat.de/, accessed on 9 September 2019). The cDNA sequence of NsLOX gene was artificially synthesized by GenScript Biochem (Shanghai, China). The synthesized NsLOX gene was digested with *Nde*I and *Xho*I, as well as inserted into pET-28a (+) (Novagen, Madison, WI, USA).

*E. coli* BL21 (DE3) (Novagen, USA) was used for gene cloning and protein expression. The *Nde*I and *Xho*I for recombinant plasmid construction were purchased from TaKaRa (Kyoto, Japan). PUFAs standards—oleic acid (OA), LA, AA, ALA, GLA, and DHA—and products of 9-HODE and 13-HODE were purchased from Merck (Darmstadt, Germany). Isopropyl β-d-1-thiogalactopyranoside (IPTG) and kanamycin (Kan) were obtained from Sangon (Shanghai, China). Tryptone and yeast extraction were purchased from Oxoid (Basingstoke, UK). The Ni^+^-NTA Agarose column (25 mL, Item code: 30210) was purchased from Qiagen (Duesseldorf, Germany) for protein purification.

### 3.2. In Silico Sequence and Phylogenetic Analysis of NsLOX Gene

The amino acid sequence of the NsLOX gene was analyzed on the ExPASy server (http://www.expasy.org, accessed on 7 October 2022). Then, the NsLOX gene was subjected to multiple sequence alignment (MSA) with LOX genes from other cyanobacterial sources via ESPcript 3.0 proram. Finally, the amino acid sequences were compared with the sequences in NCBI database by BLAST analysis, and a phylogenetic tree of LOX amino acid sequences was constructed using the N-J (Neighbor Joining) method in MEGA 5.0 software.

### 3.3. Structural Modeling of NsLOX

The three-dimensional (3D) structure of NsLOX was modeled by the SWISS-MODEL (https://swissmodel.expasy.org, accessed on 30 March 2021), with the crystal structure of a LOX from *Cyanothece* PCC 8801 (CspLOX1, PDB ID: 5EK8) as a template, which shared 26.6% primary structure similarity with NsLOX. The modeled 3D structure of NsLOX was optimized by the CHARMm force field in the Discovery Studio, and the structure that had the best geometry quality was selected further analysis. The figures of protein structure were prepared using PyMOL 2.3.0 software.

### 3.4. Recombinant Expression of NsLOX Gene in E. coli

The constructed plasmid was transformed into *E. coli*. An *E. coli* transformant harboring the recombinant plasmid pET-28a-NsLOX was named as *E. coli*/NsLOX. Single colonies of *E. coli*/NsLOX were inoculated into the 100 mL LB broth medium, supplemented with 50 μg/mL Kan, and cultured at 37 °C and 180 rpm until the OD_600_ reached around 0.6–0.8. Then, the expression of NsLOX in *E. coli*/NsLOX was induced by 100 μg/mL IPTG (0.43 mM) at 16 °C for 20 h. The cells of *E. coli*/NsLOX were collected at 8000× *g* for 5 min and stored at −20 °C [45].

### 3.5. Purification of NsLOX

The wet cells of *E. coli*/NsLOX were resuspended in the Tris–HCl buffer (20 mM, pH 8.0, 300 mM NaCl) to a wet cell. The cell suspension was disrupted by sonication, and then, the cell debris was removed by centrifugation at 8000× *g* for 30 min. The resulting supernatant was loaded on a nickel–nitrilotriacetic acid (Ni^+^-NTA) column pre-equilibrated with an equilibration buffer (20 mM Tris-HCl, pH 8.0, 300 mM NaCl, 5 mM imidazole), followed by washing with 6 column volumes wash buffer (50 mM imidazole, 20 mM Tris-HCl, pH 8.0, 300 mM NaCl) to remove impurities. The His-tagged NsLOX was eluted using the elution buffer (150 mM imidazole, 20 mM Tris-HCl, pH 8.0, 300 mM NaCl). The protein concentration was measured using Nanodrop 2000 spectrophotometer (Thermo Fisher Scientific, Carlsbad, CA, USA) at 280 nm (Abs 0.1538% = 1 g/L). SDS-PAGE (Sodium dodecyl sulfate polyacrylamide gel electrophoresis) was conducted with separating gel (12%, *w*/*v*) and polyacrylamide stacking gel (5%, *w*/*v*), which was used to analyze the purified protein.

### 3.6. Spectrophotometirc Assay of the LOX Activity

LOX activity was determined spectrophotometrically by measuring the absorbance at 234 nm, using LA as an enzyme substrate, according to the procedure described by Qian et al. [1]. The reaction mixture contained 100 μL of 2 mM LA substrate solution (at a final concentration of 66 μM), 100 µL suitably diluted NsLOX solution, and 2.8 mL Tris–HCl (20 mM, pH 8.0). The LOX activity was determined by recording the absorbance value at 234 nm within 1 min on a UV-2450 ultraviolet spectrophotometer. A single unit of LOX activity was defined as an increase in absorbance at 234 nm of 0.001 per minute.

### 3.7. Effects of pH and Temperature on the Activity and Stability of NsLOX

The optimum temperature of NsLOX was measured from 0 to 40 °C. To evaluate the thermostability, aliquots of NsLOX were treated at 4 °C to 50 °C for 5 min to 120 min prior to measuring NsLOX activity under the optimum assay conditions. The effect of pH on NsLOX activity was assayed under the optimum temperature, with the pH value ranging from 3.0 to 13.0. To evaluate the pH stability, NsLOX was incubated in different pH buffer solutions (pH 3.0 to 13.0) at 4 °C for 24 h, and then, NsLOX activity was measured under the optimum assay conditions. The highest activity was taken as 100% of the relative activity.

### 3.8. Effects of Metal Ions and Chemical Reagents on the Activity of NsLOX

To estimate the effects of metal ions (Zn^2+^, Fe^2+^, Ca^2+^, Mg^2+^, Cu^2+^, Li^2+^, Mn^2+^, and Fe^3+^) and chemical reagents (EDTA, SDS, β-Mercaptoethanol, and urea) on the activity of NsLOX, the activity was measured at a final concentration of 1 mM and 5 mM in Tris–HCl (20 mM, pH 8.0). The NsLOX activity was determined under the optimum assay conditions. The enzyme solution, without adding any additive, was used as control solution.

### 3.9. Substrate Specificity and Kinetic Parameters of NsLOX

To determine the substrate specificity of NsLOX, the specific activity of NsLOX was determined, using 2 mM OA, LA, ALA, GLA, AA, or DHA as substrate, under the optimum assay conditions. The kinetic parameters of NsLOX (0.1 mg/mL) were determined at a range of initial substrate concentrations from 20 to 100 μM of LA, ALA, or AA under the optimum assay conditions. Kinetic parameters, *K*_m_ and *V*_max_, were graphically determined from the Lineweaver–Burk plot method. The turnover number (*k*_cat_) was deduced from the *V*_max_ and the apparent molecular weight of 54 kDa. The catalytic efficiency was defined as the ratio of *k*_cat_/*K*_m_.

### 3.10. Product Analysis of NsLOX

Reaction progress was followed by recording the formation of the conjugated double bond at 234 nm. Incubations were stopped when no further increase in extinction was detectable. Hydroperoxides formed were reduced to their corresponding hydroxides with the addition of 10 mM cysteine [4]. The resulting hydroperoxy linoleic acids were extracted by adding 1 mL ethyl acetate. The extracts were dried under a continuous N_2_ stream and dissolved in methanol. The reaction mixtures were determined by LC-MS, according to the method of Zhang et al. [11]. Product specificity was evaluated by comparison with 9-HODE and 13-HODE standards.

### 3.11. Effect of NsLOX on the Quantity of Steamed Bread

NsLOX and commercial soybean flour were added to the flour at an amount of 20 U/g [42], steamed after secondary fermentation, and cooled to room temperature. The steamed bread was cut into small pieces of 2 cm^3^. The measurement was carried out by a TAXT Plus type mass spectrometer with a pre-determination rate of 1 mm/s, determination rate of 1 mm/s, and post-determination rate of 10 mm/s. The distortion tension is 50%, detection mold type is a 10 cm diameter cylindrical probe, and for the pattern data analysis, the instrument came with TAXT Texture Expert English v.1.20 software to analyze the pattern. All the measurements were conducted at least in triplicate. The one-way analysis of variance (ANOVA) was performed to evaluate the difference between means using SPSS 13.0.

## 4. Conclusions

In this work, a novel mini-LOX from *N. sphaeroides* (NsLOX) was successfully characterized. The purified NsLOX showed superior properties, including the highest catalytic activity towards LA, excellent affinity, and thermal stability over a wide pH range. Importantly, NsLOX remarkably improved the quality of steamed bread. To the best of our knowledge, the present study is the first study on the enzymatic properties of LOX from *N. sphaeroides*. Our results demonstrated that NsLOX could have great value as an excellent alternative to the current commercial LOX in the food industry. Meanwhile, NsLOX has many applications in the food industry, but it has also been associated with a few common unwanted reactions, including flavor and nutritional deterioration [6]. By studying and understanding the nature and reaction mechanism of LOX, a natural food additive, it will be better controlled and utilized.

## Figures and Tables

**Table 1:** Separation and purification steps of NsLOX.

Purification Steps	Total Activity (U)	Total Protein (mg)	Specific Activity (U/mg Protein)	Purification Fold	Yield (%)
Cell free homogenate	672,800 ± 18,013.33	408.30 ± 24.58	1647.81 ± 44.12	1	100
Nickel affinity purified NsLOX	492,216.32 ± 4814.21	6.304 ± 0.22	78,080 ± 763.68	47.38	73.16

The results are presented as mean values ± standard deviation derived from triplicate measurements.

**Table 2:** Effects of metal ions and chemical reagents on the activity of NsLOX.

Metal Ions/ Chemical Reagents	Relative Activity (%)	
	1 mmol/L	5 mmol/L
CK	100.00 ± 1.16	100.00 ± 1.26
Zn^2+^	1.39 ± 0.34	ND
Fe^2+^	76.01 ± 18.59	ND
Ca^2+^	267.91 ± 4.05	405.25 ± 5.79
Mg^2+^	139.81 ± 1.17	149.57 ± 1.50
Cu^2+^	ND	ND
Li^2+^	84.89 ± 0.66	59.92 ± 0.50
Mn^2+^	48.40 ± 1.76	ND
Fe^3+^	16.20 ± 3.43	ND
EDTA	ND	ND
SDS	ND	ND
β-Mercaptoethanol	94.96 ± 4.14	87.55 ± 1.46
Urea	91.88 ± 6.03	87.52 ± 0.88

The results are presented as mean values ± standard deviation derived from triplicate measurements.

**Table 3:** Substrate specificity of NsLOX towards six types of PUFAs.

Substrate	Structure	Relative Activity (%)
Oleic acid (OA)	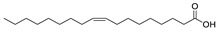	29.55
Linoleic acid (LA)	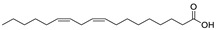	100
α-Linolenic acid (ALA)	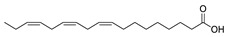	83.12
γ-Linolenic acid (GLA)	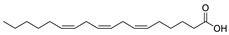	10.93
Arachidonic acid (AA)	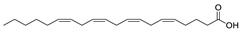	65.57
Docosahexaenoic acid (DHA)	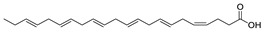	12.96

**Table 4:** The kinetic parameters of NsLOX toward LA, ALA, and AA.

Substrate	*K*_m_ (μM)	*V*_max_ (μM s^−1^)	*k*_cat_ (s^−1^)	*k*_cat_/*K*_m_ (μM^−1^ s^−1^)
LA	19.46 ± 1.24	0.64 ± 0.01	9199.75 ± 105.63	473.85
ALA	150.94 ± 6.39	0.66 ± 0.07	9394.34 ± 992.93	62.13
AA	571.22 ± 26.88	0.51 ± 0.01	7303.52 ± 144.68	12.79

The results are presented as mean values ± standard deviation derived from triplicate measurements.

**Table 5:** Texture properties of steamed bread from different experimental groups.

Gloup	Hardness (N)	Springiness (mm)	Gumminess (N)	Chewiness (mj)
Blank	9.41 ± 1.24 ^a^	9.96 ± 0.17 ^a^	6.5 ± 0.89 ^a^	64.68 ± 8.95 ^a^
Commercial LOX	8.34 ± 0.61 ^ab^	10.14 ± 0.56 ^a^	5.74 ± 0.43 ^ab^	58.09 ± 1.46 ^ab^
NsLOX	7.16 ± 0.68 ^b^	10.21 ± 0.44 ^a^	4.97 ± 0.48 ^b^	50.75 ± 5.46 ^b^

Columns with different letters indicate statistical differences (*p* < 0.05). The results are presented as mean values ± standard deviation derived from triplicate measurements.

## Data Availability

Data are contained within the article and available upon reasonable request from the corresponding author.

## Supplementary Materials

The following supporting information can be downloaded at: https://www.mdpi.com/article/10.3390/ijms24097941/s1.
