# Supplementary material for: Computational Insights and In Silico Characterization of a Novel Mini-Lipoxygenase from Nostoc Sphaeroides and Its Application in the Quality Improvement of Steamed Bread
Source: Int J Mol Sci. 2023 Apr 27;24(9):7941. doi: 10.3390/ijms24097941 (PMC10177866; doi:10.3390/ijms24097941)
Supplement: Supplementary file 1 [file ijms-24-07941-s001.zip › ijms-2269615-supplementary.pdf]

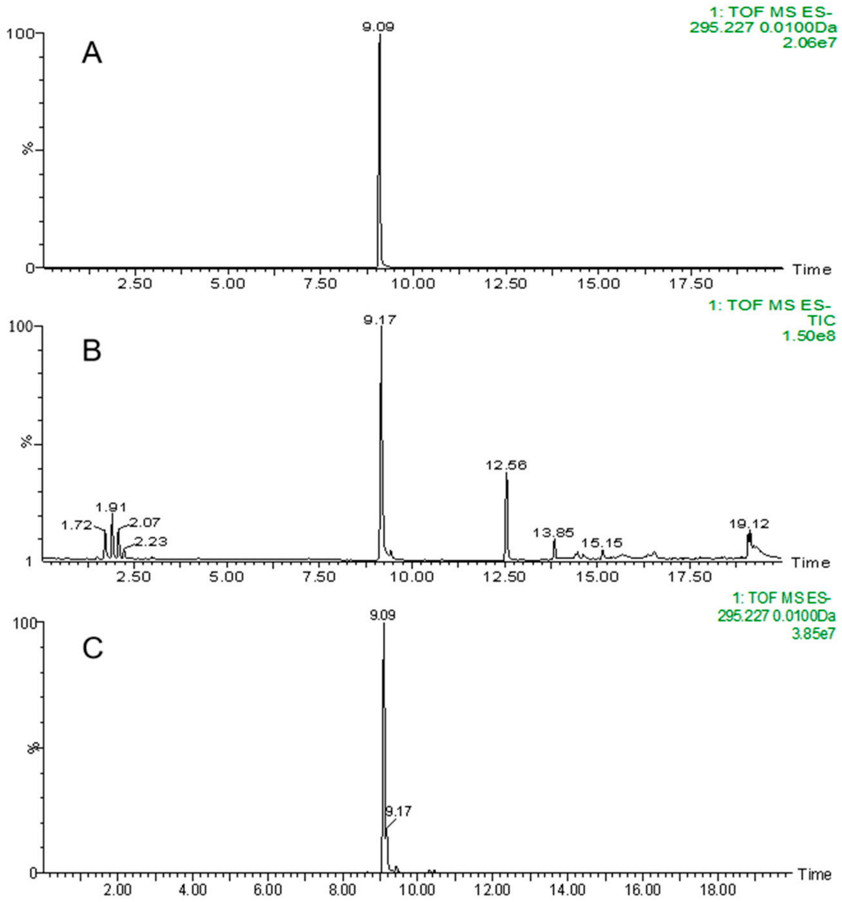

Figure S1. Identification of the product obtained from LA by the reaction of NsLOX. (A) LC-MS profile of the standard 9-HODE. (B) LC-MS profile of the standard 13-HODE. (C) LC-MS profile of the product.
